# PRIME - A multilevel intervention for low back pain management in primary care: Protocol for an effectiveness-implementation hybrid type 1 cluster randomized controlled trial

**DOI:** 10.1016/j.conctc.2026.101679

**Published:** 2026-08-12

**Authors:** G. Christe, B. Darlow, J. Hartvigsen, J. Stanic, M. Rosa, J. Virdee, M. Martel, M. Lötscher-Stamm, S. Wieser, D. Schaller, O. Kherad, S. Genevay

**Affiliations:** a HESAV School of Health Sciences - Vaud, HES-SO University of Applied Sciences and Arts Western Switzerland, Lausanne, Switzerland; b Department of Primary Health Care, University of Otago Wellington, Aotearoa, New Zealand; c Center for Muscle and Joint Health, Department of Sports Science and Clinical Biomechanics, University of Southern Denmark, Odense, Denmark; d Chiropractic Knowledge Hub, Odense, Denmark; e Danish Institute of Advanced Study, University of Southern Denmark, Odense, Denmark; f Research Institute of the McGill University Health Centre, Montreal, Canada; g Winterthur Institute of Health Economics, ZHAW Zurich University of Applied Sciences, Winterthur, Switzerland; h Faculty of Health Sciences and Medicine, University of Lucerne, Lucerne, Switzerland; i Réseau Delta, Vésenaz, Switzerland; j Internal Medicine Division, Hôpital de La Tour and University of Geneva, Geneva, Switzerland; k Department of Rheumatology, Geneva University Hospitals, Geneva, Switzerland

**Keywords:** Low back pain, Primary care, Exercise, Reassurance, Education, Adoption, Stratified care

## Abstract

**Background:**

Low back pain (LBP) is a leading cause of disability and healthcare utilization worldwide, yet management in primary care often remains inconsistent with clinical guidelines, with underuse of high-value care and persistent use of low-value interventions. The primary objective of this study is to evaluate the effectiveness of PRIME (PRImary care MultilEvel intervention for low back pain) compared with usual care in terms of cost-effectiveness (system level), back-related imaging rates (practitioner level), and self-reported disability (patient level), while concurrently examining implementation processes.

**Methods:**

This study uses an effectiveness–implementation hybrid type 1 design (NCT07137065). Effectiveness will be evaluated through a pragmatic cluster randomized controlled superiority trial conducted in primary care in Switzerland, recruiting 500 patients with LBP from February 2026 to June 2027. General practitioner quality circles will be randomized to PRIME or usual care. PRIME combines clinician training, patient education resources, automated decision support, and a stepped care pathway aligned with patients’ risk of chronicity. Implementation outcomes and determinants will be assessed in the intervention group using quantitative and qualitative methods guided by established implementation frameworks.

**Discussion:**

This trial will provide a comprehensive evaluation of a multilevel intervention targeting both clinical and implementation gaps in LBP care. By integrating effectiveness and implementation outcomes, the study aims to generate actionable evidence on how high-value LBP care can be sustainably implemented in routine primary care and inform future scale-up strategies.

## 1 Introduction

Low back pain (LBP) is the leading cause of disability worldwide and represents a substantial burden for healthcare systems, particularly in primary care settings [1]. In Switzerland, 50% of people report recurrent LBP, which is associated with work limitations, and increased healthcare utilization [2]. Despite well-established clinical guidelines [[3], [4], [5]], the management of LBP in primary care remains highly variable, with underuse of high-value care, and overuse of low-value care strategies [6,7]. Therefore, effective strategies are needed to improve the integration of guideline-concordant care in primary care [8].

Unhelpful beliefs about LBP, such as thinking that the back is fragile and that LBP is caused by serious injury, are frequently identified among healthcare professionals [9,10], patients with LBP [11] and the general population [12,13]. These beliefs are associated with maladaptive behaviors and reduced use of high-value care [14,15]. They are also associated, together with other psychological factors, with an increased risk of chronic disabling LBP [16]. Moreover, clinicians report limited confidence and lack of practical tools to identify and address these psychological risk factors in daily practice [17,18]. Interventions that support clinicians to address these factors early and to align care with patients’ risk of chronicity may improve recovery and reduce unnecessary investigations and costs [19,20].

Supporting clinicians to integrate best practice recommendations requires more than education alone. Multilevel, theory-informed interventions co-designed with stakeholders, adapted to local contexts, and supported by implementation strategies such as reminders, decision-support tools, audit and feedback are more likely to lead to sustainable behavior change [[21], [22], [23], [24]]. Effectiveness–implementation hybrid designs have been recommended to simultaneously evaluate clinical effectiveness and implementation processes, while optimizing research efficiency [[25], [26], [27]].

The PRImary care MultilEvel intervention for low back pain (PRIME Back Study) was co-developed by clinicians, LBP experts and people with lived experience of LBP to improve the integration of high-value care in primary care through a coordinated, multilevel strategy. The intervention was designed to help general practitioners (GPs) and physiotherapists to address psychological risk factors of chronicity, reduce low-value care and increase high-value care strategies.

The primary objective of this effectiveness-implementation hybrid trial is to evaluate the effectiveness of PRIME compared with usual care at three levels: cost-effectiveness (system-level); imaging prescription rate (practitioner-level), and self-reported back-related disability (patient-level). These three distinct, complementary primary outcomes will facilitate a rigorous evaluation to ascertain whether it is advisable to implement the PRIME intervention. Cost-effectiveness was selected as the system-level primary outcome because demonstrating value for money is essential for health policy decisions in LBP care [28]. The imaging prescription rate was selected as the practitioner-level primary outcome because it is a direct, objective indicator of guideline-concordant GP behavior and a key target of PRIME's clinician-facing components [29]. Self-reported back-related disability was selected as the patient-level primary outcome because it is recommended as a core outcome in LBP research [30]. Usual care in this trial reflects standard primary care practice in a healthcare network in French-speaking Switzerland, where GPs already participate in regular quality circle meetings and have unrestricted access to specialist referral, physiotherapy, and interdisciplinary rehabilitation. This high-quality, ecologically valid comparator was chosen to demonstrate the added value of PRIME over existing care. Secondary objectives include evaluating the effectiveness of PRIME on opioid and sick leave prescriptions, clinicians' beliefs and confidence in treating patients with LBP (practitioner-level), as well as pain intensity, work participation, health-related quality of life, global perceived change, pain self-efficacy, fear avoidance beliefs, and beliefs about LBP (patient-level). In addition, implementation processes and outcomes will be examined among healthcare practitioners, including acceptability, appropriateness, feasibility, adoption, fidelity, and barriers and facilitators to implementation. Finally, patients' experience and acceptability of PRIME will be investigated qualitatively.

## 2 Methods

### 2.1 Trial context and design

This study employs an effectiveness-implementation hybrid type 1 design, in which clinical effectiveness is the primary focus while implementation processes are evaluated as secondary objectives [[25], [26], [27]]. Effectiveness will be evaluated through a pragmatic, cluster-randomized controlled superiority trial comparing PRIME with usual care at system, practitioner and patient levels. The 20 quality circles (clusters) will be randomly allocated to the intervention or control group at a 1:1 ratio (10 intervention clusters, 10 control clusters). All clusters are drawn from a single healthcare network (“Réseau Delta”) in French-speaking Switzerland. Within this network, GPs take part in regular quality circles—small groups of about five to ten physicians—that offer a structured setting for peer exchange and continuous practice improvement. Implementation processes will be investigated using quantitative and qualitative methods [25,26].

This study was approved by the Regional Ethic Committee (CER-VD, 2025-01380). Healthcare practitioners participating in the study and all patients signed an informed consent. The study is registered on clinicaltrials.gov (NCT07137065). Patient recruitment is planned from February 2026 to June 2027.

### 2.2 Patient and public involvement

One person living with LBP is a member of the core research team and contributed to the development of the intervention and study design, including selection of outcome measures, educational materials, and study procedures. He will also participate in data interpretation and dissemination.

### 2.3 Eligibility criteria

#### 2.3.1 Healthcare professionals

Clusters will consist of quality circles of general practitioners (GPs) from the Réseau Delta. Quality circles will be eligible if their GPs practices are located within 20 min by car or 30 min by public transport from an interdisciplinary management clinic. GPs will be eligible if they belong to an included quality circle, provide informed consent, and complete the pre-study training (2 h for the intervention group; 30 min for the control group). To minimize contamination, if GPs from the same practice belong to two different quality circles, only those belonging to one of these circles will be eligible. Physiotherapists will be eligible if they complete the two-day PRIME training and have facilities for active therapy. Occupational therapists will be eligible if they work in an interdisciplinary management clinic participating in the study. Health professionals are eligible to take part irrespective of their level of clinical experience.

#### 2.3.2 Patients

Patients aged ≥18 years consulting a participating GP for a primary complaint of LBP (with or without leg pain) will be eligible. There is no upper limit on LBP duration, reflecting the heterogeneity of LBP presentations in primary care. Pain duration at baseline will be recorded. Exclusion criteria include consultation with their GPs for LBP within the previous three months; inability to provide informed consent; inability to access or complete French-language electronic questionnaires; pregnancy; lumbar surgery within the past six months; or confirmed specific spinal pathology (e.g. inflammatory disease, malignancy, infection, fracture, or cauda equina syndrome). Patients will also be excluded if they present with a major motor deficit defined as grade ≤ 3 on the Medical Research Council Scale for muscle strength, indicating movement possible against gravity but not against resistance, or if they require immediate referral to emergency or specialist care [31]. As specific causes of LBP may not be identifiable at initial consultation, patients with a subsequent confirmed diagnosis of specific spinal pathology may be excluded post allocation. The rate of post-randomization exclusion due to confirmed specific spinal pathology is expected to be approximately 1–3% of enrolled patients and is not anticipated to differ systematically between study groups [19]. All post-randomization exclusions will be performed by a blinded medical doctor and reported in the CONSORT flow diagram.

### 2.4 Recruitment procedures

#### 2.4.1 Healthcare professionals

**Fig. 1:**
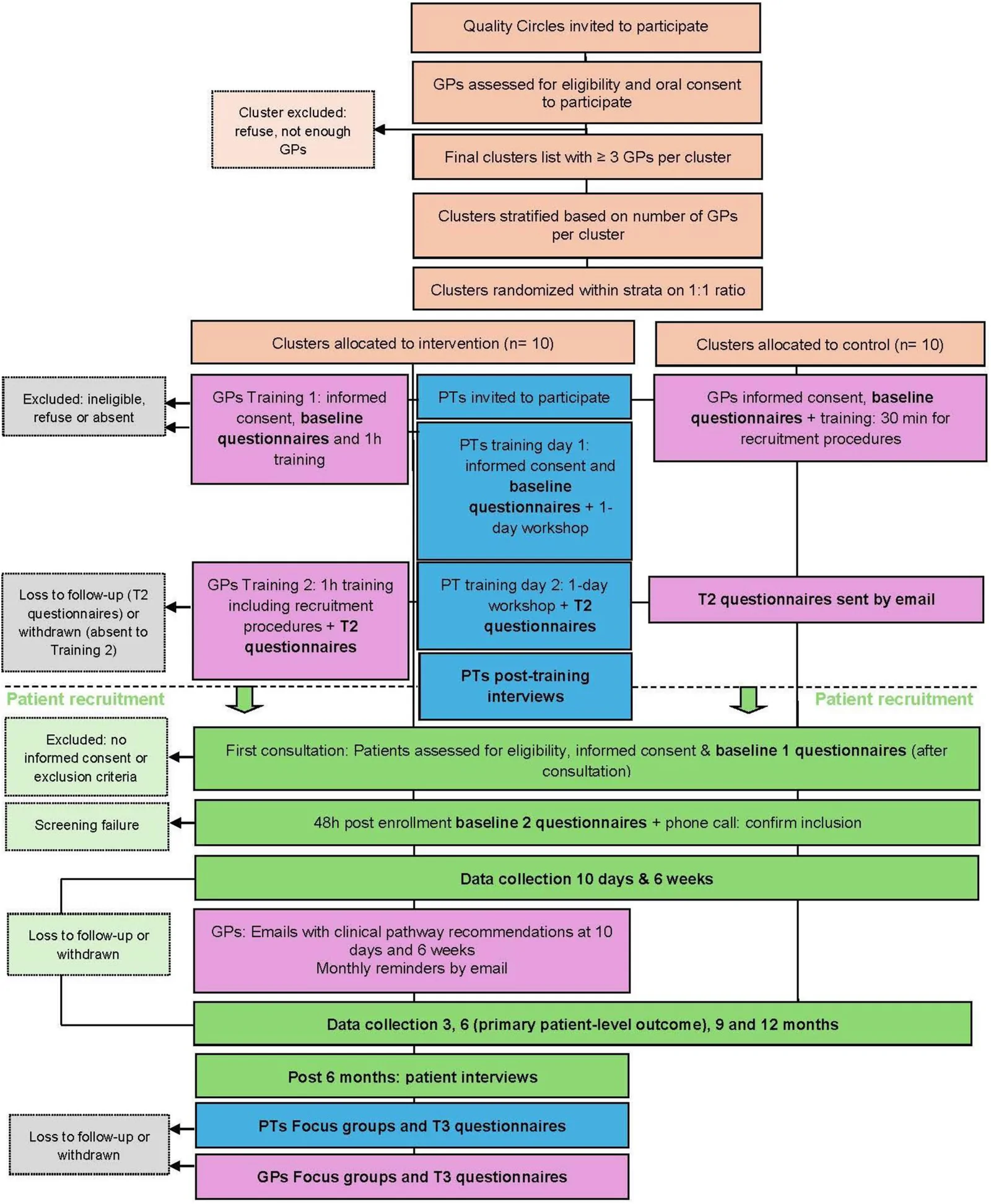
Study participation flow diagram GPs: general practitioners; PTs: physiotherapists; Pink: related to GPs; Blue: related to PTs; Green: related to patients.

Quality circles will be recruited from the Réseau Delta in French-speaking Switzerland (Fig. 1). Eligible quality circles will be identified and contacted through the network coordination team. Quality circles in which at least three GPs agree to participate will be included prior to randomization, ensuring that GPs and the research team remain blinded to group allocation during recruitment. Small quality circles (≤4 GPs) will be merged with another geographically proximate quality circle before randomization to form a single cluster. Additional GPs may be added to randomized clusters if they provide consent after randomization. GPs will provide written informed consent at the start of the pre-study session.

Physiotherapists practicing in the geographical area of intervention clusters will be recruited after cluster randomization through GP referral, direct contact by the research team, and professional social media channels. Occupational therapists will be recruited from interdisciplinary management clinics affiliated with the study network.

#### 2.4.2 Patients

Patient recruitment will occur after cluster randomization of clinicians, with patients allocated according to their GP's study arm. All patients consulting a participating GP for LBP will be screened during the first consultation and provided with study information. Eligible patients who consent will enroll by scanning a QR code, which triggers baseline questionnaires and notifies the research team. The research team will then contact patients by phone to confirm understanding of study procedures and address questions. Patients who withdraw consent following this contact or fail to complete baseline questionnaires will be considered screening failures and excluded, with all collected data deleted.

To prevent and monitor selection bias, objective enrolment criteria will be provided to GPs in advance, and GPs will be asked to log all screened patients using the QR code, including reasons for non-inclusion. Practices will receive CHF 30 per included patient to support administrative workload, and regular monitoring of recruitment will be conducted to support enrolment and assess selection bias.

### 2.5 Interventions

#### 2.5.1 Intervention group

PRIME is a multilevel intervention designed to promote guideline-concordant, high-value care for LBP in primary care. It comprises core clinical components targeting GP consultations, physiotherapy management, interdisciplinary rehabilitation, and patient education, supported by various implementation strategies (Table 1). It was developed in accordance with the Medical Research Council guidance for complex interventions [25]. Content and intervention components were informed by international clinical guidelines [3,4,32], systematic reviews [15,17,29,33], and evidence from clinical trials [34,35] addressing low back pain management in primary care. The intervention was co-developed by a multidisciplinary research team in collaboration with GPs, physiotherapists, and occupational therapists to ensure clinical relevance and feasibility [25]. PRIME was piloted in primary care, and qualitative feedback from clinicians was used to refine intervention components and delivery procedures prior to this trial (in publication).

**Table 1:** Multilevel intervention.

	Core clinical components	Implementation strategies
**GPs**	**GP (first) consultation(s)** EXPLORE • Risk factor of chronicity • Perform a physical exam • Use active listening EXCLUDE emergency cases during the first consultation • Infection • Osteoporotic fracture • Severe neurological issues EXPLAIN - REASSURE • Build confidence in movement • Keep explanations simple • Use educational resources* • LBP Booklet • Website for patients PRESCRIBE • Recommend movement and gradual return to activities • Work absence to a minimum If needed: • Physiotherapy • Medication	2 x 1-h interactive training during two remunerated quality circle meetings, facilitated by opinion leader. For each hour, GPs received 200CHF. In addition: • Between-training practice • Intervention manual • Website for GP • Patient videos Monthly reminders Patient education resources*
	**Clinical pathway recommendations** At 10 days and 6 weeks, clinical pathway recommendations are based on SBT level of risk and capacity to work, as well as PT reports if the patient has already started PT.	Automatic emails at 10 days and 6 weeks. Leaving choice and responsibility to GP in conjunction with PRIME
**PTs**	**Management strategies** EXPLORE • Explore beliefs • Explore motor behaviour • Validate and build relationship EXPOSE • Movement strategies modifications • Gradual exposition to feared movements and activities • Exercise variation EXPLAIN • Patient centered explanation • Check the understanding (teach back method) • Shared decision on objectives EMPOWER • Increase load and progressive exercises • Physical activity self-management • Management of flare-up	2 × 1 day workshop, facilitated by opinion leader, including: • Between-training practice • Intervention manual • Website for PT • Patient videos • Supervised treatment with simulated patients Follow-up online session Checklist for self-assessment Leaving choice and responsibility to PT (they are free to integrate other approaches in conjunction with PRIME)
**IR**	**Assessment of risk factors** • One-two individual sessions with a specialized OT to confirm the need for IR and provide target education • Rehabilitation program: individual and group sessions with OTs and PTs, focusing on education and active exercises.	GP training and resources to facilitate access to IR if needed.

**Fig. 2:**
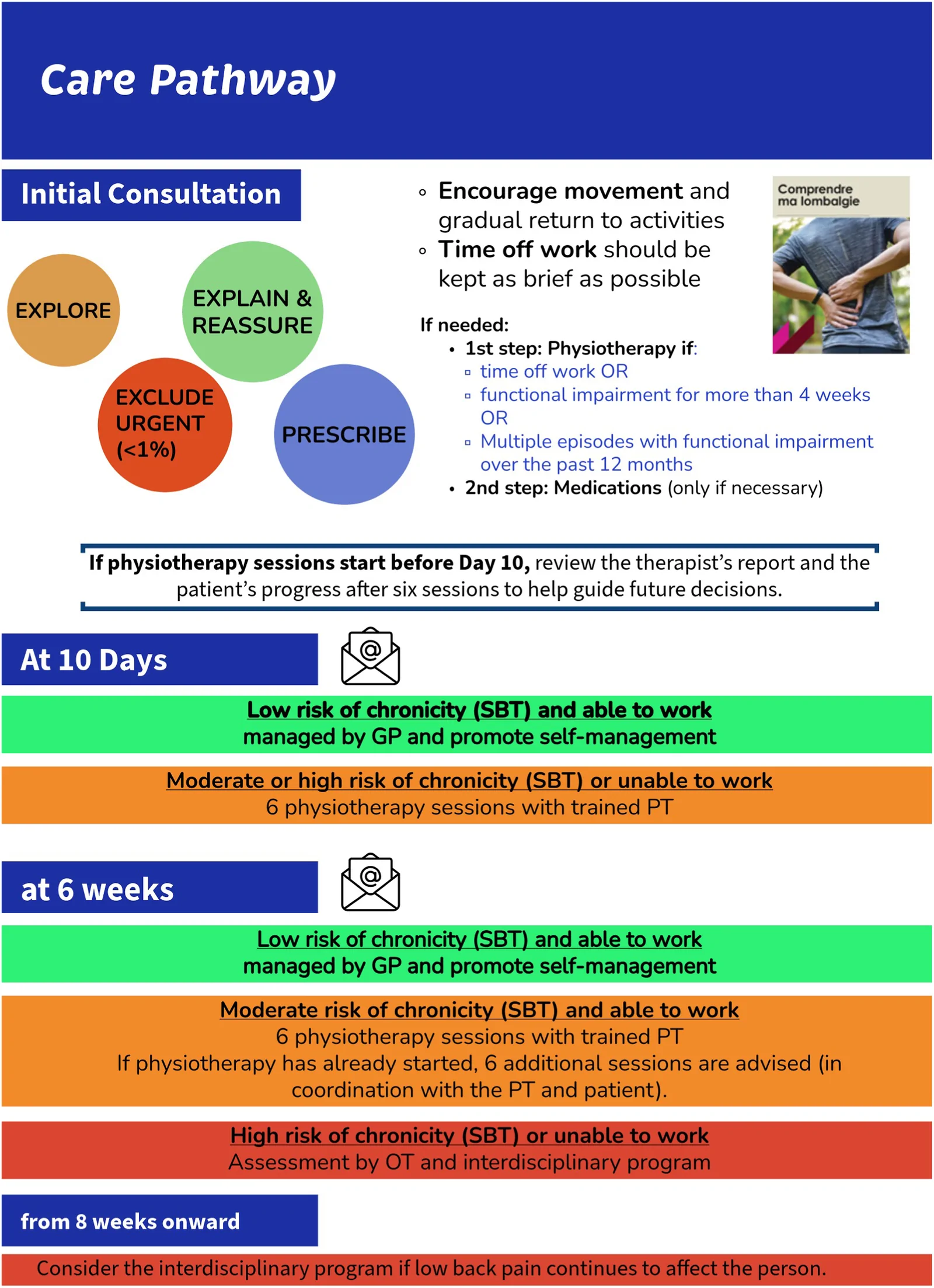
Care Pathway SBT: Start Back Tool; GP: General practitioner; PT: physiotherapist; OT: occupational therapist.

Core components of PRIME include (1) training for GPs to support evidence-based assessment, reassurance, and risk-stratified care [23,33]; (2) training for physiotherapists to deliver biopsychosocial, guideline-concordant rehabilitation that target risk factors of chronicity [[36], [37], [38]]; (3) structured patient education resources [35,39,40]; and (4) a stepped clinical care pathway supported by automated decision support linked to patients’ risk of chronicity and interprofessional communication, including access to trained physiotherapists and interdisciplinary rehabilitation if needed (Fig. 2) [41,42]. Implementation strategies include interactive training sessions, educational materials, reminders, and automated clinical decision support tools.

GP training is delivered at the cluster level during quality-circle meetings, while physiotherapists complete a two-day workshop prior to delivering the intervention. Patient education and pathway-based care are initiated during the GP consultation, with escalation based on risk of chronicity criteria. Full details of the intervention and implementation strategies are reported in a TIDieR checklist (Supplementary Material S1), with detailed descriptions of GPs and physiotherapists training provided in Supplementary Materials S2 and S3.

Between 6 weeks and 3 months after the start of patient recruitment, GPs in the intervention group will be contacted by email to provide feedback on the intervention. All modifications will be documented and reported. Adaptations are anticipated to be minor and operational (e.g., timing of reminder emails, interprofessional communication procedures) and do not constitute changes to the clinical content of PRIME, the randomization scheme, or the primary outcome definitions. A formal adaptive trial design was not adopted, as the scope of planned adaptations does not require pre-specified interim analyses or sample size re-estimation.

#### 2.5.2 Control group

GPs in the control group will receive no specific training or intervention. They will provide usual care, which includes unrestricted access to physiotherapy, imaging, medication, sick leave, and specialist referral (including interdisciplinary rehabilitation) as clinically indicated. This reflects real-world Swiss primary care practice and is considered the best choice comparator. A 30-min information session on recruitment procedures is provided to ensure comparable enrolment processes without contaminating usual care. After all follow-up data has been collected from participants recruited within a control arm quality circle, that quality circle will be offered the same training as received by those in the intervention group.

#### 2.5.3 Concomitant care

Participants in both groups may receive any additional care (concomitant care) at the discretion of their GP or themselves, including physiotherapy, imaging, medications, and specialist consultations. Concomitant care is distinct from the study intervention. All concomitant care for LBP will be recorded at each follow-up time point via patient self-report questionnaires, and complemented where available with health insurance data.

GPs: General practitioners; PTs: physiotherapists; IR: interdisciplinary rehabilitation; *Patient education resources are present in the core clinical components (goal: improve patients' beliefs which should influence clinical outcomes – eg. reduce pain & disability) and the implementation strategies (goal: intervening on patients’ beliefs can enhance the uptake and adherence of the intervention amongst GPs).

### 2.6 Outcomes

The primary system-level outcome is the incremental cost per quality-adjusted life-year (QALY), estimated using the EQ-5D-5L, incorporating costs from both a healthcare payer and a societal perspective [43]. Direct medical costs over 12 months will be collected through a structured patient-reported healthcare utilization questionnaire administered at each follow-up time point, capturing GP consultations, physiotherapy, specialist visits, investigations (imaging, blood tests), medications, injections, surgery, and hospitalization, previously used in Switzerland [28]. Where available, self-reported data will be complemented and validated using health insurance reimbursement data provided by the main healthcare insurances in Switzerland, covering patients insured by this provider. Indirect costs related to absenteeism and presenteeism will be assessed using the Work Productivity and Activity Impairment Questionnaire (WPAI) [44,45].

The primary practitioner-level outcome is the proportion of patients receiving back-related imaging within 6 months of the initial consultation [29,35]. Secondary practitioner-level outcomes include opioid prescription, medication use, imaging rates and total number over 12 months, clinicians’ beliefs about LBP (measured with the 10-item Back Pain Attitudes Questionnaires, Back-PAQ), and confidence in managing LBP [[46], [47], [48], [49]].

The primary patient-level outcome is back-related disability measured by the 24-item Roland–Morris Disability Questionnaire (RMDQ) at 6 months [50]. Secondary patient-level outcome measures include pain intensity (Numerical Rating Scale, NRS), health-related quality of life (EQ-5D-5L), work participation (WPAI), Global Rating of Change (11-point scale, GRC), pain self-efficacy (Pain Self-Efficacy Questionnaire short form, PSEQ-2), a range of psychological factors, including Fear of movement, Catastrophizing, Anxiety, Depression (Brief screening questions from Kent et al., 2014, BSQ), expectations to return to work (one question from Orebro questionnaire), beliefs about LBP (10-item Back-PAQ) and reassurance (Reassurance Questionnaire) [43,46,[51], [52], [53], [54]]. Based on the advice of our patient partner, two questions were added about capacity to perform valued activities and confidence in their back. PSEQ-2, BSQ and Back-PAQ questionnaires will help investigate the mechanisms of action of the PRIME intervention.

Adverse events (AEs) will be monitored through both passive and active surveillance. Passive surveillance involves continuous self-reporting by participants to the research team or their treating GP. Active surveillance is conducted through REDCap, where participants are prompted to report health events; any declaration triggers an automated alert to the research team for evaluation. Serious AEs are defined as events resulting in death, hospitalization or in persistent disability and are not anticipated given the non-invasive, non-pharmacological nature of the intervention. The expected non-serious AE is a fluctuation or increase in back or leg pain symptoms; this type of event is sometimes referred to as a normal reaction and will not be reported by GPs or physiotherapists.

Healthcare professionals baseline data will include general characteristics such as age, gender, years of clinical experience, practice setting and type, and prior training in LBP management. Patient baseline data will include demographics and social determinants of health. Participants will receive online questionnaire links by SMS and email. To limit participant burden, baseline data will be collected over two timepoints: on the day of the initial GP consultation and two days later. Follow-up assessments will occur at 10 days, 6 weeks, and 3, 6, 9, and 12 months (Supplementary Material S4). To promote retention, non-respondents will receive up to three reminders, followed by telephone contact if needed. Participants may receive financial compensation of up to CHF 50 based on questionnaire completion.

### 2.7 Implementation outcomes and determinants

Implementation outcomes and determinants will be assessed among patients and healthcare professionals in the intervention group only, guided by the Theoretical Domains Framework (TDF), the Consolidated Framework for Implementation Research (CFIR), the theoretical framework of acceptability (TFA), and Proctor et al.’s conceptual model [[55], [56], [57]]. Quantitative and qualitative methods will be used, as detailed in Table 2.

**Table 2:** Implementation outcomes.

Outcome	Data Source	Timeline	Framework
Healthcare professionals			
Acceptability Appropriateness Feasibility	Acceptability of Intervention Measure (AIM) Questionnaire Intervention Appropriateness Measure (IAM) Questionnaire Feasibility of Intervention Measure (FIM) Questionnaire	GPs (IG) & PTs: At the end of training, after patient recruitment. OTs: After patient recruitment only	Proctor
	Focus Group with GPs, PTs and OTs	After patient recruitment	TFA, Proctor
Adoption *Proportion of recruited patients managed according to PRIME by GPs*	Patient self-report in REDCap and data from healthcare insurances: 1. Alignment of GP decision with treatment pathway recommendation 2. Rate of imaging and medication prescription 3. Work absence prescription 4. Rate of booklet use: “Did your doctor used resources (information booklet, website, drawings, …) to explain my low back pain during the last consultation? *Response: yes/no + free text to describe it*	1-3. Over the recruitment period 4. Baseline 2	Proctor
	Focus Group with GPs	After patient recruitment	Proctor
Adoption *Proportion of patients having physiotherapy sessions managed according to PRIME by PTs*	Patient self-report in REDCap: • Did the patient see a trained PRIME PT? PT checklist: • To what extent did you use the PRIME intervention with this person? *Response: 0: Not at all; 1: A little; 2: Moderately; 3: A lot (Adoption considered for 2 or 3 points)*	Over the recruitment period	
	Focus Group with PTs	After patient recruitment	Proctor
Fidelity - GPs *The degree to which the intervention is delivered as intended.*	GPs: Indirect measure through patient self-report in REDCap: 1. Reassurance: To what extent did the physician … - Reassure you that he/she had no serious concerns about your back - Explain how the treatment offered would help with your problem *Response: 0 (Not at all) - 6 (Extremely). > 3 was considered as fidelity* 2. LBP beliefs and Fear avoidance - Back-PAQ score - FABQ score	Baseline 2	Proctor
	Focus Group with GPs	After patient recruitment	Proctor
Fidelity - PTs *The degree to which the intervention is delivered as intended.*	PT checklist • To what extent did you explore beliefs (using reflections, open-ended questions, etc.)? • To what extent did you explore problematic functional movements? • To what extent did you modify movement, increasing muscle relaxation, speed, and amplitude ? • To what extent did you expose the person to functional movements that were feared and/or painful for them? • To what extent did you use load and speed in the exercises? • To what extent did you use a variety of exercises during the sessions ? • To what extent did you use explanations to reassure the person that were centered on their history and physical examination? • To what extent did you discuss returning to activities, using pacing and the gradual increase of physical activities? • To what extent did you propose strategies for managing new episodes/flare-up ? *Response: 0: Not at all; 1: A little; 2: Moderately; 3: A lot (Fidelity considered for 2 or 3 points)*	At the end of physiotherapy treatment sessions	Proctor
	Focus Group with PTs	After patient recruitment	Proctor
Perception of biopsychosocial competencies amongst PTs	Semi-structured interviews	After training	TDF; CFIR
Barriers and Facilitators of PRIME in clinical practice	Semi-structured interviews with PTs	After training	TDF; CFIR
	Focus group with GPs, PTs and OTs	After patient recruitment	

Patients			
Acceptability and experience	Satisfaction with the intervention, collected through patient self-report in REDCap: • To what extent were you satisfied with the care provided by your general practitioner for your back pain? • To what extent were you satisfied with the care provided by your physiotherapist for your back pain? • To what extent were you satisfied with the care provided by the interdisciplinary program for your back pain? Responses: Very satisfied*; Rather satisfied*; Neither satisfied nor dissatisfied; Rather dissatisfied; Very dissatisfied (acceptability in *)	Patients (both arms)	Proctor
	Narrative interviews	After 6-month primary outcome collection in the IG only	TFA; CFIR

GP: general practitioner, PT: physiotherapist; OT: occupational therapist; IG: intervention group; in grey, qualitative data collection.

Quantitative data will be collected through self-report questionnaires completed by healthcare professionals and patients in both groups. Qualitative data will be collected through focus groups (FG) (or semi-structured interviews if required) with GPs, physiotherapists, and occupational therapists after completion of patient recruitment [58,59]. Interviews will also be conducted with physiotherapists at the end of training and with patients in the intervention group after the 6-month primary outcome assessment to explore their experiences and acceptability of PRIME. Sampling will aim for diversity in professional roles, patient trajectories, and exposure to PRIME components. The final number of FG and interviews will be determined based on information power and availability of the participants [60]. FG and interview guides will be informed by implementation frameworks, as well as previous research and results from the pilot study whenever relevant [57,58].

### 2.8 Sample size

The sample size was calculated based on the primary patient-level outcome (RMDQ) and the primary practitioner-level outcome (imaging prescription rate) [61]. Assuming 10 clusters per group with a mean of 20 patients per cluster, the study has 80.97% power to detect a between-group difference of 2.5 points on the RMDQ (SD = 6.0), using an intracluster correlation coefficient (ICC) of 0.05 and a two-sided significance level of 0.05 [62]. The ICC of 0.05 and the 20% allowance for loss to follow-up were informed by Darlow et al. (2019), who used the same assumptions in a comparable cluster-randomized primary care study evaluating the RMDQ. For back-related imaging, the same cluster structure provides 81.99% power to detect a 15% absolute reduction from a baseline imaging rate of 30%, assuming an ICC of 0.01 [35,63]. Allowing for a 20% dropout rate, a total of 500 patients will be recruited (25 patients per cluster).

Variation in cluster size was assessed using the coefficient of variation approach [64]. Given a minimum of 15 and a maximum of 35 patients per cluster (mean = 25), the coefficient was < 0.23 and considered negligible. Therefore, no adjustment was applied, and recruitment will stop once a cluster reaches 35 patients. No formal sample-size calculation was performed for cost-effectiveness due to limited Swiss data. We aim to recruit at least 100 general practitioners (approximately five patients per GP) and a minimum of 30 physiotherapists.

### 2.9 Randomization

The 20 clusters will be randomly allocated to the intervention or control group (1:1). Allocation concealment is ensured by the use of a computer-generated random sequence held by a blinded statistician, with cluster allocation unknown to recruiters prior to randomization. Randomization will be stratified by cluster size (small vs large, defined by the median number of GPs per cluster) to ensure balance between study arms.

### 2.10 Blinding

Due to the nature of the intervention, investigators responsible for delivering the PRIME training (GC, SG) will be aware of GP cluster allocation but will not be involved in data collection, data management, or statistical analyses. In case of a serious adverse event or medical condition, the PI may contact participants while aware of allocation, but any decision regarding participant exclusion will be made by a blinded medical doctor.

Outcome data will be entered directly by patient participants via Research Electronic Data Capture (REDCap) [65]. Research staff contacting participants for questionnaire reminders will be blinded to group allocation. REDCap access for data collection staff is configured to exclude cluster and group identifiers. Reminder contacts follow standardized scripts that do not reference intervention components or care received, and patients are unaware of their group allocation. Staff conducting qualitative interviews will be aware of allocation, as interviews will be restricted to participants in the intervention group. Therefore, interviews will be conducted after completion of the 6-month primary outcome assessment.

GPs and physiotherapists will be aware of their allocation due to intervention delivery but will not be involved in data collection or analysis. Patients will remain unaware of their group allocation through limited disclosure and will be informed only that the study evaluates care pathways for LBP. Patients in the intervention group may possibly infer their allocation through receipt of PRIME components (e.g., patient education booklet). However, this is inherent to complex behavioral interventions and is acknowledged as a limitation.

Data cleaning and statistical analyses will be performed by a statistician blinded to group allocation. The analysis dataset will not include cluster or group identifiers, and results will be unblinded only after completion of analyses.

### 2.11 Data collection and management

Patient- and practitioner-reported data will be collected and managed using REDCap and securely stored on Geneva University Hospital (HUG) servers protected by institutional firewalls. Data modifications will be fully traceable within REDCap. Identifiable information will be stored separately from coded study data in distinct REDCap projects.

Data not collected via REDCap (e.g. qualitative data and administrative cost data) will be stored on secure university servers. Access to all study data will be limited to authorised research team members using institutional access control and password protection. All study data will be coded automatically in REDCap.

### 2.12 Statistical methods

Cost-effectiveness will be evaluated as the incremental cost-effectiveness ratio (ICER), expressed as net cost in CHF per quality-adjusted life-year (QALY) gained. QALYs will be derived from the EQ-5D-5L assessed at baseline and at 3, 6, and 12 months, using the French EQ-5D-5L value set [66], as there is currently no value set for Switzerland. QALYs will be estimated using the area-under-the-curve method over the 12-month follow-up, meaning that QALYs are calculated by multiplying the patients’ health state utility scores with the time spent in that health state.

Costs from the healthcare payer perspective will be estimated from self-reported healthcare utilization and, where available, health insurance data, applying current Swiss tariffs (TARDOC). The societal perspective will additionally include productivity losses due to absenteeism and presenteeism, assessed using the WPAI. Costs and QALYs will not be discounted given the one-year time horizon. Uncertainty will be assessed using non-parametric bootstrapping to estimate 95% confidence intervals for the ICER, and probabilistic sensitivity analyses will be conducted [67,68]. Cost-effectiveness will be explored across a range of willingness-to-pay thresholds.

Practitioner and patient-level outcomes will be analyzed according to the intention-to-treat principle by a blinded statistician. Primary and secondary continuous outcomes will be analyzed using linear mixed-effects models with random intercepts for clusters to account for the hierarchical structure of the data (patients nested within quality circles). Adjusted models will also be performed based on baseline variables (e.g. age, gender, socio-economic health determinants, RMDQ). Categorical outcomes (e.g. imaging prescription) will be analyzed using generalized linear mixed models [69].

For patient-level primary outcome, an exploratory non-inferiority analysis will be performed using a margin of −2.5 points on the RMDQ, reflecting the possibility that the intervention may be acceptable if non-inferior and cost-saving. Pre-planned mediation analyses will examine the role of psychological factors in patient outcomes [70]. Different secondary analyses will be conducted, including the number of participants in each group that reach 30%, 50% and 70% of improvement on the RMDQ, as well as the number of participants reaching the patient acceptable symptom state (≤4 on RMDQ) [71]. All analyses will be performed using SAS (version 9.4) and Stata or R. Deviations from the statistical analysis plan will be documented and reported.

Given the three primary outcomes operating at distinct and complementary levels (system, practitioner, patient), findings will be interpreted jointly but are considered complementary rather than causally dependent. Each outcome is analyzed and reported independently, without formal alpha correction across levels. Discordant findings across levels are anticipated and theoretically meaningful: the three outcomes cover different domains serving different decision-making purposes, consistent with the GRADE Evidence to Decision framework [72].

Participants who withdraw or fail to complete baseline questionnaires will be considered screening failures and excluded from the trial. To ensure sufficient power at the primary endpoint, recruitment will continue until at least 200 patients per group have completed the 6-month assessment; consequently, total recruitment may exceed 500 participants. GP dropouts during the study period will not result in patient exclusion. Patient outcomes will be analyzed according to the cluster allocation at enrolment *(intention-to-treat principle)*.

For cost-effectiveness analyses, missing cost data will not be imputed; instead, they will be complemented where available with health insurance records. In case of discordance between data sources, patient questionnaire data will be considered primary and insurance data will be used for validation and to complement missing values. For outcomes other than costs, multiple imputation assuming data Missing at Random will be performed using SAS PROC MI for variables with more than 10% missing data, incorporating covariates, intervention group, and cluster information.

### 2.13 Qualitative data analysis

All focus groups and interviews will be audio-recorded, transcribed verbatim, and analyzed using Reflexive Thematic Analysis (RTA) [73]. Although RTA favors inductive coding, deductive coding based on implementation frameworks (e.g., TDF, CFIR) may be employed, especially when pertaining to specific implementation processes and determinants [57,73,74]. Two team members will participate in data generation and analysis. In line with current rigor standards in qualitative health research, initial analysis will be independently performed by two researchers on a subset of the data to form an initial codebook. Coding will be performed using MAXQDA software [75]. Analytic disagreements will be resolved through discussion and consensus, with a third researcher consulted if needed.

### 2.14 Monitoring and amendments

The trial will be monitored in accordance with ICH-GCP E6(R3) using a proportionate, risk-based approach. Off-site monitoring will be conducted before study initiation, during the first 3 months and then a minimum of once per year, or more frequently if triggered by safety signals, monitoring findings, or significant recruitment deviations. Monitoring will be performed by an independent qualified monitor not involved in trial conduct. The monitor will review essential documentation, informed consent records, eligibility, REDCap data quality, and adverse event reporting. Monitoring findings will be documented and corrective actions overseen by the principal investigator. Substantial protocol amendments require written approval from the ethics committee before implementation.

### 2.15 Dissemination

Study results will be published in international journals and presented at conferences. Findings will also be disseminated to healthcare professionals through training activities involving GPs, physiotherapists, and occupational therapists. Results will be shared with patients and the public via media, social media, conferences, and information websites. The patient partner will be involved in dissemination activities.

## 3 Discussion

PRIME was designed as a multilevel intervention targeting modifiable psychological and behavioral risk factors of chronicity along the patient care pathway. PRIME also includes implementation strategies to support the adoption and sustainability of the intervention. By addressing risk factors of chronicity and management decisions across professional and patient levels, PRIME aims to maximize its impact on both healthcare utilization and patient-reported outcomes.

Previous studies have shown inconsistent results of risk-based stratification with the STarT Back Tool (SBT) [34,76,77]. In PRIME, we tried to address limitations from prior research: SBT applied at Day 0 may over-identify psychological distress due to transient acute reactions, while assessment at Day 10 improves prognostic accuracy [78]. Combining SBT with work capacity extends stratification beyond psychological risk alone [41], and the automatization process requires less consultation change and effort from GPs. Unlike the original SBT trial, which primarily targeted GP referral behavior, PRIME simultaneously intervenes on GP practice, physiotherapy competencies, and patient education. Finally, co-development with Swiss primary care clinicians and pilot study refinement are expected to support ecological validity and uptake of the SBT.

PRIME was co-developed with clinicians and refined through a pilot study, with particular attention to feasibility and acceptability of PRIME. Feedback from healthcare professionals informed adaptations before the start of this trial, in line with the MRC framework [25], and is expected to support uptake in routine practice.

This study uses an effectiveness–implementation hybrid design to evaluate PRIME across multiple levels, including cost-effectiveness, practitioner behavior, and patient outcomes. Given that patient outcomes in primary care often improve over time regardless of intervention, assessing system-level and practitioner-level effects is critical for understanding the broader value of PRIME. Concurrent evaluation of implementation outcomes will help to explain how and why the intervention succeeds or fails and inform future scale-up strategies.

Several risks and limitations should be considered. First, patient recruitment is led by GPs, which may introduce selection bias despite mitigation strategies. Second, recruitment may also be challenging given GP workload, even though we included a high number of GPs in the study and developed recruitment strategies with a low time burden. If recruitment targets are not achieved, the recruitment period may be extended and additional eligible clusters may be recruited. If the final sample size remains below target, this will result in greater imprecision of the effect estimates and an increased risk of type II error, which will be acknowledged in the limitations. Third, the geographically limited setting increases the risk of contamination, particularly if patients in the control group consult trained physiotherapists. This will be monitored through patient questionnaires that will capture whether control-arm participants consulted a PRIME-trained physiotherapist. In such cases, sensitivity analyses will be conducted to assess the impact on primary outcomes. GP-level contamination is minimized by excluding GPs from the same practice who belong to different quality circles, and by restricting intervention materials (training content, digital tools, reminder emails) to intervention clusters only. The risk that control GPs will be passively exposed to intervention components in a meaningful way is low, and the risk that any exposure leads to important practice change to be even less [19]. Fourth, baseline data were collected after the initial GP consultation, which may limit evaluation of early consultation effects. Fifth, although LBP prevalence in primary care is not known to vary substantially by season, temporal factors such as GP availability during holiday periods or staggered training schedules could influence recruitment rates. Cluster-level recruitment monitoring will be conducted throughout the enrolment period. Finally, the effectiveness of complex behavioral interventions depends on fidelity and engagement, which may vary across clinicians, and co-interventions cannot be fully controlled. Accordingly, this trial primarily evaluates the effectiveness of interventions targeting healthcare professionals rather than individual clinical encounters.

## Declaration of use of generative AI

AI-assisted writing support (Microsoft Copilot and Claude) was used to improve language clarity and readability. The authors assume full responsibility on all intellectual content of this manuscript.

## Sources of funding

This trial is funded by the 10.13039/100000001Swiss National Science Foundation (Grant: PT0043_222873).

## CRediT authorship contribution statement

**G. Christe:** Conceptualization, Funding acquisition, Methodology, Project administration, Supervision, Writing – original draft. **B. Darlow:** Conceptualization, Methodology, Resources, Writing – review & editing. **J. Hartvigsen:** Conceptualization, Methodology, Resources, Writing – review & editing. **J. Stanic:** Conceptualization, Methodology, Writing – review & editing. **M. Rosa:** Investigation, Methodology, Writing – review & editing. **J. Virdee:** Conceptualization, Resources, Writing – review & editing. **M. Martel:** Conceptualization, Methodology, Resources, Writing – review & editing. **M. Lötscher-Stamm:** Methodology, Writing – review & editing. **S. Wieser:** Methodology, Writing – review & editing. **D. Schaller:** Resources, Writing – review & editing. **O. Kherad:** Resources, Writing – review & editing. **S. Genevay:** Conceptualization, Methodology, Resources, Supervision, Writing – review & editing.

## Declaration of competing interests

The authors declare the following financial interests/personal relationships which may be considered as potential competing interests:Guillaume Christe reports financial support was provided by Swiss National Science Foundation. If there are other authors, they declare that they have no known competing financial interests or personal relationships that could have appeared to influence the work reported in this paper.

## Data Availability

No data was used for the research described in the article.
