# Supplementary material for: PRIME - A multilevel intervention for low back pain management in primary care: Protocol for an effectiveness-implementation hybrid type 1 cluster randomized controlled trial
Source: Contemp Clin Trials Commun. 2026 Aug 12;53:101679. doi: 10.1016/j.conctc.2026.101679 (PMC13499054; doi:10.1016/j.conctc.2026.101679)
Supplement: Supplementary file 1 [file mmc1.docx]

| **TIDieR item** | **Core elements of PRIME (Innovation)** | **Implementation strategies** |
| --- | --- | --- |
| **1. Brief name** | PRIME Back | |
| **2. Why (rationale)** | PRIME aims to improve the integration of high‑value care for LBP in primary care by addressing key mechanisms underlying the persistence of disability and the use of low‑value care. The intervention is based on three complementary rationales:   1. Psychological and behavioural factors play a central role in the transition from acute to persistent low back pain. Unhelpful beliefs, fear of movement, and avoidance behaviours are modifiable risk factors for chronicity and disability and therefore constitute primary targets of the intervention. 2. Education and promotion of movement are evidence‑based first‑line strategies for LBP management. PRIME prioritises reassurance, meaningful education, and graded return to activity as core clinical strategies, rather than passive treatments or unnecessary investigations. 3. Unhelpful beliefs must be targeted at multiple levels to achieve sustained change. PRIME therefore addresses beliefs and behaviours not only in patients, but also among general practitioners and physiotherapists, recognising that clinicians’ beliefs influence clinical decisions, patient communication, and care pathways. | Implementation strategies are designed to support adoption, fidelity, and sustainability of PRIME in routine primary care. They target known barriers to guideline implementation, including limited clinician confidence in addressing psychological factors, time constraints, and reliance on habitual practice patterns. Strategies focus on enhancing clinicians’ capability, motivation, and opportunity to deliver PRIME through training, decision support, reminders, and integration within existing clinical structures. |
| **3. What (Materials)** | Patients will receive an education booklet *“Understanding My Low Back Pain”* that showed promising results in reducing the use of imaging has been translated into French and adapted to the Swiss context [1, 2]. Patients will also be directed to the website [www.infomaldedos.ch](http://www.infomaldedos.ch), which provides consistent evidence-informed messages on LBP [3]. | GPs also receive supporting material to promote change in practice, including a booklet that summarizes the intervention, access to a website for healthcare practitioners with videos of model clinical interactions. The patient booklet is also used as a facilitating strategy to increase patient education during the first consultation.  PTs will receive a comprehensive intervention manual, PDF of presentations and access to a website with multiple resources, including videos of model clinical interactions specific to physiotherapists. |
| **4. What (Procedures)** | **General practitioners**  GP consultations including triage, reassurance, education, and pathway‑based referral, which includes PT management with trained PTs and escalation to interdisciplinary programme when indicated.  GP consultations include:  EXPLORE  •Risk factor of chronicity  •Perform a physical exam  •Use active listening  EXCLUDE focus on emergency cases during the first consultation  •Infection  •Osteoporotic fracture  •Severe neurological issues  EXPLAIN - REASSURE  •Build confidence in movement  •Keep explanations simple  •Use educational resources  PRESCRIBE  •Recommend movement and gradual return to activities  •Work absence to a minimum  If needed, during the first encounter:  •Physiotherapy  •Medication  At 10 days and 6 weeks, clinical pathway recommendations are sent automatically to the GP by email, based on SBT level of risk and capacity to work, as well as PT reports if patient has already started PT.  If the risk is low and the patient is able to work, care is managed by GP and self-management is promoted. At 10 days, patients with moderate or high risk (SBT) or unable to work should receive 6 physiotherapy sessions with a trained PT. A 6 weeks, trained PT is also prescribed in case or moderate risk of chronicity if the person is able to work. If there is still a high risk of chronicity or the patient is not able to work at 6 weeks, an interdisciplinary program is advised.  **Physiotherapy management strategies**  EXPLORE   - Explore beliefs - Explore motor behaviour - Validate and build relationship   EXPOSE   - Movement strategies modifications - Gradual exposition to feared movements and activities - Exercise variation   EXPLAIN   - Patient cantered explanation - Check the understanding (teach back method) - Shared decision on patient objectives   EMPOWER   - Increase load and progressive exercises - Physical activity self-management - Management of flare-up   Physiotherapists will also send a short report at the end of six physiotherapy sessions to the GP.  **Interdisciplinary care**  Before the interdisciplinary care is started, 1-2 assessment sessions with an OT are planned to explore a diversity of contributing factors and assess the relevance of interdisciplinary program.  Interdisciplinary care is composed of PT individual sessions, OT individual sessions, and group sessions that target physical and psychological factors of back pain through education and exercises. | **General practitioners**  2 x 1-hour interactive training during two remunerated quality circle meeting, facilitated by opinion leader. These two training sessions are separated by a least one month. In addition, GPs receive instructions for between-training practice, with access to all the available resources.  During the trial, they receive monthly reminders with information on PRIME.  GPs receive for each patient at 10 days and 6 weeks an automated email with clinical pathway recommendation. Based on discussion with GPs, previous research and our pilot work, it was critical that the clinical decision support is automated to limit clinicians’ burden. The email will only communicate the current state of the patient and the GP is free to decide whether to follow the clinical pathway recommendation, as independence of practice is also important for implementation.  **Physiotherapists**  2 x 1 day workshop, facilitated by opinion leader, including between-training practice. During the workshop, they had time to practice all aspects of physiotherapy management. They also performed supervised treatment with simulated patients.  Follow-up online sessions will also be programmed. They will also receive monthly reminder with information on PRIME and interprofessional communication.  When they see a patient with LBP for the PRIME trial, they will have to record their treatment in a checklist. While the checklist is primarily designed for fidelity measurement, it will also serve as a reminder of practice.  To facilitate implementation, PT have the choice and responsibility to use PRIME and other interventions in conjunction. |
| **5. Who provided** | General practitioners are part of the Delta Network, a care network in which they meet 10 times per year in quality circle to improve their practice. All general practitioners in the intervention group received 2x1 hour of training.  Physiotherapists can be included in the study if they have participated in the two-day training workshop, and have a space for active therapy in their clinic. There were no restriction in previous experience or training.  Occupational therapists and physiotherapists working in Rachis Clinic centers will provide the interdisciplinary care. They did not receive any training for the study. | GP training will be provided by a rheumatologist with extensive LBP expertise will provide the training, given that opinion leaders can facilitate behavior change.  PT training was provided by an expert physiotherapist, with extensive clinical and teaching experience of psychologically-informed physiotherapy. |
| **6. How (mode of delivery)** | GPs have all a first session with their patient in the practice. Following sessions are optional and to the discretion of the GP.  PT will receive a prescription for 6 individual sessions of physiotherapy. Additional sessions can be asked if the patient is progressing, but objectives are not completed.  Interdisciplinary care is composed of assessment sessions, PT individual sessions, OT individual sessions, and group sessions with active exercises. | For GPs, the two 1-hour training sessions were held during a quality circle meeting, online or in person, depending of the uses of the quality circle. At least one month separate the two training sessions. Reminders are sent at least once a month by email.  The two-day workshop was held in person, with around 20-25 PTs attending the course. The tow days are separated by a two months period. Reminders are sent by email. Online meeting are proposed to the PT in case of questions. |
| **7. Where** | GPs have all a first session with their patient in their practice. Following encounters, if needed, can be in person, by phone or email, depending of the practice of the GP.  PTs will see their patient in their practice that includes a space for active therapy.  Interdisciplinary care will be held in one center of the Rachis clinic, an interdisciplinary structure that provides structures programs for spinal-related conditions. | For GPs, training sessions were held during a quality circle meeting, online or in person, depending of the uses of the quality circle.  The two-day workshop was held in person in one large PT center, or at the university. |
| **8. When and how much** | For GPs, the intervention will be delivered during routine consultations. No minimum of maximal umber of sessions are planned. Physiotherapy typically initiated as a 6‑session block with possible escalation (up to 18 sessions). Interdisciplinary care is composed of 2 assessment sessions, 6 PT individual sessions, 6 OT individual sessions, and 12 group sessions. | GP training was delivered during 2x 1-hour training sessions before the start of the trial. Reminders were sent every month during the recruitment period.  Physiotherapy training was 2 in‑person days separated by practice period before the start of the trial. Monthly reminders are sent during the recruitment period. |
| **9. Tailoring** | The care pathway was tailored to patient risk of chronicity and work capacity. All interventions can be adapted by healthcare practitioners to the need of the patient. We deliberately leave room for adaptation to the healthcare practitioners, as independence of practice is important for implementation of new practice. | Both GP and PT training and reminders were planned, but allow to adapt to the need and questions of the individuals. |
| **10. Modifications** | Minor adaptations to the intervention are planned according to healthcare practitioners feedback during the first months of the intervention. All modifications will be documented. | Timing and content of reminder and decision‑support emails may be adjusted in response to clinician feedback. All modifications will be documented. |
| **11. Fidelity (planned)** | We plan to assess GP fidelity indirectly, through patients’ responses (e.g. uses of education resources, reassurance) and administrative data (imaging and medication prescription).  Pt fidelity will be assessed through a self-reported checklist. | We will monitor all training sessions and reminders to assess fidelity of implementation strategies. |
