# Supplementary material for: PRIME - A multilevel intervention for low back pain management in primary care: Protocol for an effectiveness-implementation hybrid type 1 cluster randomized controlled trial
Source: Contemp Clin Trials Commun. 2026 Aug 12;53:101679. doi: 10.1016/j.conctc.2026.101679 (PMC13499054; doi:10.1016/j.conctc.2026.101679)
Supplement: Supplementary file 2 [file mmc2.docx]

| **Interventions** | **Rationale** | **TDF** → COM-B | **BCTs** | **ERIC** |
| --- | --- | --- | --- | --- |
| **GP training** |  |  |  |  |
| Two 1-hour of interactive education sessions | Discuss current GP practice and recommendations in a group meeting, allowing to compare one own's practice to best care [1] | **Knowledge, Skills, Beliefs about capabilities** → Capability (Psychological & Physical), Motivation (Reflective) | 4.1 Instruction on how to perform behaviour; 5.1 Information about health consequences; 6.1 Demonstration of behaviour;  15.1 Verbal persuasion about capability | Conduct educational outreach visits; Make training dynamic |
| Education sessions facilitated by opinion leader | Increase credibility and acceptance [1] | **Social influences, Social/professional role & identity** → Opportunity (Social), Motivation (Reflective) | 9.1 Credible source;  6.2 Social comparison |  |
| Meetings within usual quality circle meeting | Fit into existing practice and no extra burden [6] | **Environmental context & resources** → Opportunity (Physical) |  | Use existing structures; Tailor strategy (pilot study) |
| Remuneration | Remuneration is part of the Delta Network support for continuing education [6] | **Reinforcement** → Motivation (Automatic) | 10.1 Material incentive | Fund and contract for the clinical innovation |
| Time to practice between sessions | Reinforce skill acquisition between educational meetings [3] | **Skills, Behavioural regulation** → Capability (Psychological & Physical) | 8.1 Behavioural practice; 8.3 Habit formation; 8.7 Graded tasks | Conduct ongoing training |
| **GP educational resources** | |  |  |  |
| Intervention guide | Provide structured support for clinical practice [3] | **Knowledge, Memory/attention, Behavioural regulation** → Capability (Psychological) | 4.1 Instruction on how to perform behaviour; 6.1 Demonstration of behaviour; 7.1 Prompts/cues | Develop and distribute educational materials |
| Website | Provide accessible information and video demonstrations [3] | **Knowledge** → Capability (Psychological) | 4.1 Instruction on how to perform behaviour; 6.1 Demonstration of behaviour | Develop and distribute educational materials |
| **Reminders** |  |  |  |  |
| Monthly reminders | Ongoing reminders on PRIME principles [1;2;3] | **Memory, attention & decision processes; Behavioural regulation** → Capability (Psychological) | 7.1 Prompts/cues; 8.3 Habit formation; 1.5 Review goals | Remind clinicians |
| **Patient education resources** | |  |  |  |
| Patient education booklet | By reducing unhelpful beliefs among patients, and using tools to explain why imaging is not needed, GPs will have less pressure from the patients to request imaging [4;5;6] | **Environmental context & resources, Social influences, Beliefs about consequences** → Opportunity (Physical), Opportunity (Social), Motivation (Reflective) | 12.5 Adding objects to environment; 5.1 Information about health consequences; 5.6 Information about emotional consequences; 9.1 Credible source | Intervene with patients/consumers to enhance uptake and adherence; Tailor strategy (pilot study) |
| Patient education website |  |  |  |  |
| **Clinical decision tools** |  |  |  |  |
| Automatic email with treatment recommendation | Automated support decision-making, without burden for the GP [1;6] | **Memory/attention; Environmental context & resources; Behavioural regulation** → Capability (Psychological), Opportunity (Physical) | 7.1 Prompts/cues;8.1 Behavioural practice; 12.5 Adding objects to environment | Remind clinicians; Tailor strategy |
| Leaving choice and responsibility to GP | GPs keep control on clinical decisions. They are free to decide what is best for the patient, acknowledging their competencies [6] | **Social/professional role & identity; Beliefs about capabilities; Beliefs about consequences** → Motivation (Reflective) | 15.1 Verbal persuasion about capability; 13.2 Framing/reframing | Promote adaptability |

BCTs: Behaviour change techniques; ERIC: Expert Recommendations for Implementing Change

[1]: Chauhan et al. (2017). https://doi.org/10.1186/s13012-016-0538-8

[2]: Tzortziou Brown et al. 2016. DOI: 10.1002/14651858.CD007495.pub2.

[3]: Mesner et al. (2016). https://doi.org/10.1186/s12891-016-1110-z

[4]: Johnson et al. (2015). https://doi.org/10.1136/bmjopen-2015-008592

[5]: Slade et al. (2016). https://doi.org/10.1097/AJP.0000000000000324

[6]: Lau et al. (2015). https://doi.org/10.1186/s13012-016-0396-4
