# Supplementary material for: PRIME - A multilevel intervention for low back pain management in primary care: Protocol for an effectiveness-implementation hybrid type 1 cluster randomized controlled trial
Source: Contemp Clin Trials Commun. 2026 Aug 12;53:101679. doi: 10.1016/j.conctc.2026.101679 (PMC13499054; doi:10.1016/j.conctc.2026.101679)
Supplement: Supplementary file 3 [file mmc3.docx]

**PRIME PT training**

Contents

[1.	Training Description:	1](#_Toc185583320)

[2.	Learning objectives:	1](#_Toc185583321)

[3.	Description of the Physiotherapist Intervention for Patients with Low Back Pain	2](#_Toc185583322)

[4.	Training Plan with Integrated Objectives	3](#_Toc185583323)

[5.	Reporting	5](#_Toc185583324)

[6.	References	8](#_Toc185583325)

1. Training Description:

This two-day training program aims to develop the skills of physiotherapists to manage low back pain using a psychologically-informed and graduated activation approach. A key focus is on assessing and addressing unhelpful patient beliefs about pain and movement, reducing kinesiophobia and avoidant behaviour, and promoting a positive and functional view of the back. Participants will learn practical strategies to implement in physiotherapy practice to shift patients' perceptions, encourage engagement in valued activities, and foster long-term behavioural change.

The training emphasizes experiential learning, with dedicated workshops on communication skills and active behavioural exercises, such as graded exposure and individualized exercise programs.^1–3^ Through role-playing, case-based discussions, practice and examples of individualized and functional exercises, participants will gain competency in targeting cognitive and behavioural factors in their practice. The two days in person are separated to allow application of skills from the first day in clinical practice before attending the second day. During this time, participants have access to a website with multiple resources, including videos of PRIME intervention with a patient. A follow-up mentoring ensures ongoing application and competency in real-world settings.

2. Learning objectives:

After the 2-day courses, participants will be able to:

1. Describe the multifactorial nature of low back pain.

2. Perform a person-centred interview that investigate person understanding, goals and expectations.

3. Perform a patient-centred physical examination including behavioural experiments.

4. Perform a behavioural and active treatment based on the principles of graded exposure to reduce kinesiophobia and encourage the return to functional activities.

5. Demonstrate good communication skills to target unhelpful beliefs and promote a shared and positive understanding of back problems.

6. Provide an individualized exercise program that includes functional exercises, progressive loading of the spine and variations.

7. Apply structured clinical reasoning to integrate psychosocial factors into therapeutic decision-making.

3. Description of the PRIME PT Intervention for Patients with Low Back Pain

The PRIME PT intervention is designed to address modifiable psychological and behavioural factors that contribute to the persistence of low back pain, including kinesiophobia, unhelpful beliefs, avoidant behaviour, and low self-efficacy. These factors have been shown to be important treatment mediators that should be targeted in rehabilitation by physiotherapists.^4–6^

**Intervention Structure: PRIME PT**

The intervention is organized around four core pillars: Explore, Expose, Explain, Empower (4E), which guide the physiotherapist's approach to assessment and treatment:

*1. Explore: Building Understanding and Alliance*

- During the initial interview and physical examination, the physiotherapist explores the patient’s beliefs, fears, and behaviours regarding their back pain using motivational interviewing techniques and behavioural experiment. A particular attention in the physical examination is dedicated to unhelpful motor behaviour, such as reduced amplitude and velocity of movement and muscle guarding.

- The patient’s experience is validated, and a strong therapeutic alliance is created to foster trust and collaboration.

*2. Expose: Encouraging Positive Experiences with Movement*

- Patients are progressively exposed to movements and activities to create new, positive experiences that build confidence in their ability to move without harm, based on the finding of ‘*Explore’*.

- Principles of safety learning, inhibitory learning theory, and expectancy violation are applied to co-construct helpful beliefs and increase the confidence that movement is safe.^8–10^

- Graded exercises are used to gradually build confidence with movement and physical activity.

*3. Explain: Fostering a Shared Understanding*

- Through dialogue and exposure, the physiotherapist develops a shared understanding of the patient’s condition, addressing misconceptions and promoting a positive, biopsychosocial perspective of their back pain.

- Explain focuses on explaining pain mechanisms, discuss beliefs that are not aligned with achieving their goals, and reinforcing the idea that movement is safe and beneficial.

- Explain is strongly related to the person’s story or physical examination. Brief and simple messages are used to help the person to make sense of their back pain.

*4. Empower: Enhancing Self-Efficacy, behaviour change and Management Skills*

- The intervention prioritizes increasing the patient’s self-efficacy and confidence by providing variations in exercises and incorporating different contexts for practice.

- Patients are supported in developing self-management skills, including flare-up management plans, to reduce dependence on the therapist and build long-term resilience.

- Exercises are individualized, functional, and progressive, with a focus on empowering patients to re-engage in meaningful activities.

**Key Outcomes**

The intervention seeks to help patients change their behaviour by:

- Strengthening beliefs aligned with a positive, functional view of their back.

- Reducing kinesiophobia and avoidant behaviours through safe, graded exposure to movement.

- Building confidence in managing their pain and physical activity independently.

- Developing the skills and strategies necessary to prevent setbacks and maintain long-term progress.

By combining these pillars, the intervention provides a comprehensive, patient-centred approach that integrates psychological, behavioural, and physical rehabilitation for low back pain.

4. Training Plan with Integrated Objectives

| Phase | Objective(s) | Content | Teaching strategies and planning |
| --- | --- | --- | --- |
| Day 1 Morning (8h30-12h30): Integration of psychological and behavioural factors in the assessment. | 1. Explain the current understanding of low back pain. 2. Conduct a thorough interview in a patient-centred manner. 3. Conduct a physical examination including behavioural experiments. | - Review of intervention principles.  - Screening Tool (SBT)  - Techniques for patient-centred interviews. - Behavioural experiments during physical exam, focussed on motor behaviour strategies | **1. Group introduction** **30’** (introduction: informed consent; questionnaires);  **2. LBP understanding – general theoretical introduction 45’**  **3. Clinical case study (30’)**: case study to identify psychological and behavioural factors.  BREAK  **4. Interview principles (20’) & Role-Playing (30’):** Introduction and practice of interviews in pairs using scenarios, with trainer feedback.  **5. Physical examination (1h):** Trainer show behavioural experiments and motor behaviour assessment. Supervised practice with trainer feedback. |
| Day 1 Afternoon (13h30-17h):  Gradual exposure, exercises progression, key messages | 4. Demonstrate a behavioural and active treatment based on the principles of graded exposure to reduce kinesiophobia and encourage the return to functional activities. 5. Demonstrate good communication skills to target unhelpful beliefs and promote a shared and positive understanding of back problems. | - Principles of graded exposure and movement modification. - Strategies to address unhelpful beliefs and build patient understanding. - Techniques for promoting positive perspectives on movement and pain. | **6. Gradual Exposure Practice and movement modification (1h):** Participants design, and practice graded exposure plans based on the clinical case study.  **7. Exercises progression and variation (1h):** Trainer demonstration and participant practice of exercise progression and variation.  BREAK 15’  **8. Discuss key messages to develop a shared understanding (45’):** Discuss in group the main messages we can use to promote a positive view of movement and the back. Practice Explain.  **9. Day conclusion (30’):** presentation of tasks for the period until day 2; checklist |
| Clinical Practice Period (2 Months) | 4. Demonstrate a behavioural and active treatment based on the principles of graded exposure to reduce kinesiophobia and encourage the return to functional activities.  5. Demonstrate good communication skills to target unhelpful beliefs and promote a shared and positive understanding of back problems. 6. Demonstrate an individualized exercise program that includes functional exercises, progressive loading of the spine, and variations.  7. Apply structured clinical reasoning to integrate psychosocial factors into therapeutic decision-making. | - Participants implement graded exposure, active treatment strategies, and patient education in clinical practice. - Focus on integrating individualized exercise programs and self-management strategies. | **Support Options:** 1. A PRIME Checklist for reflecting on cases. 2. Trainers available via email for case-specific questions.  3. Website:  - Articles  - Videos of PRIME PT (Explore, Expose, Explain)  - Intervention manual |
| Day 2 Morning  (8h30-12h30): | 2. Conduct a thorough interview in a patient-centred manner.  4. Demonstrate a behavioural and active treatment based on the principles of graded exposure to reduce kinesiophobia and encourage the return to functional activities.  5. Demonstrate good communication skills to target unhelpful beliefs and promote a shared and positive understanding of back problems. 7. Apply structured clinical reasoning to integrate psychosocial factors into therapeutic decision-making. | - Discuss clinical experiences: challenges, successes, and insights. - Advanced skills in communication and building therapeutic alliances. - Integrating psychosocial and motor factors into structured clinical reasoning.  - recognition of referral to the multidisciplinary program. | **1. Welcome, description of the day and feedback by participants on post-it (15’)**  **2. Description of PRIME Trial and role of PT (30’)**  **3. Empower (45’): presentation and discussion:** self-management strategies, flare-up management, self-efficacy  BREAK 20’  **4. Clinical case study (1h10):** Participants discuss in group a case of LBP with radicular pain. They propose a management plan according to PRIME. Discussion on the role of manual therapy and how to manage radicular pain.  **5. Briefing for the simulated patient session in the afternoon (30’)** |
| Day 2 Afternoon  (13h30-17h): | 5. Demonstrate good communication skills to target unhelpful beliefs and promote a shared and positive understanding of back problems.  6. Demonstrate an individualized exercise program that includes functional exercises, progressive loading of the spine, and variations. | 1. Designing and implementing functional, progressive exercises. 2. Educating patients on self-management. - Wrap-up: Creating an action plan for continued skill application. | **6. Simulated Patients (3h):** Participants treat simulated patients under trainer and group supervision, focusing on active interventions and education. Main aspects of PRIME developed: EXPLORE, EXPOSE, EXPLAIN  BREAK 20’  **Planning for next months (30’):** Discussion to prepare next months’ activities; answering questions. T2 questionnaires |
| Post-Training Follow-Up | all | - Continued support: Trainers provide feedback and monitor progress if needed. - Participants present updated cases in follow-up group calls (1–2 months later). - Encourage reflective practice. | - **Virtual Follow-Up:** Schedule group calls to discuss cases and answer questions  - **Self-Evaluation:** Use a checklist to assess integration of PRIME PT in practice. |

6. Reporting

Following the recommendations of Simpson et al. (2021),^2^ here is a structured reporting of the training program:

**1. Defining the Physiotherapist Intervention**

- **Content and Components:** The training focuses on psychological and behavioural dimensions of low back pain management. See intervention description (above) for details. Yet, physiotherapists are allowed to use any other techniques or strategies in addition to the intervention described above.
- **Dose:** The first GP prescription includes six 30-minute sessions. Recommended distance between sessions: 1 week. If patient is not progressing, he/she should be sent for review at the interdisciplinary team. Maximal total number of sessions: 18.
- **Scope of Practice:** The training emphasizes evidence-based strategies that fall within the scope of physiotherapy, such as communication techniques, risk of chronicity assessment, investigation of beliefs and goals, behavioural exercises, and patient education. In the intervention, patients that have started the physiotherapy intervention but still have high risk of chronicity and/or high level of pain or disability, will be referred to a interdisciplinary program with comprehensive evaluation of psychosocial factors. The possible referral to a psychologist or psychiatrist will be made by the multidisciplinary team or the GP.

**2. Training Towards Competency**

- **Trainers:** The trainer is an experienced physiotherapist with expertise in psychologically-informed and graded exposure approaches. He has also pedagogical (more than 15 years’ experience) and has research expertise on this topic.
- **Content:**
  - Understanding low back pain (biopsychosocial approach).
  - Addressing kinesiophobia, unhelpful beliefs, and avoidant behaviours with psychologically-informed physiotherapy. These include:
    - Patient interview in a person-centred approach
    - Gradual exposure
    - Motivational Interviewing techniques
    - Movement modification (increase flexion, relaxation, speed)
    - Functional, progressive, varied and individualized exercises (with load)
    - Management of physical activity (pacing, graded activity)
  - Communication techniques to promote a positive perspective on movement. Different examples of patient education.
  - Radicular pain management
  - Red flags – how to recognize specific pathologies
  - Role of manual therapy in the management of low back pain
- **Format/Structure:**
  - **Day 1 training:** Workshops focused on assessments, interviews, and initial treatment planning.
  - **Clinical Practice Period:** Participants apply skills in their clinical settings with optional trainer support. Website with multiple resources.
  - **Day 2:** Advanced practical sessions, clinical reasoning based on real patients’ cases and simulated patient interactions.
  - **Follow-Up:** Virtual group mentoring and competency review.
- **Dose:**
  - Day 1 and Day 2: 7 hours each.
  - Clinical practice period: 8 weeks of independent application.
  - Follow-up mentoring: Minimum 1 online session of 60 minutes. Individual contact as requested.
- **Approach to Skill Development:** Training emphasizes experiential learning through role-playing, practical workshop, supervised patient simulations, and reflective discussions. Skills are reinforced in clinical practice between the two in-person workshop and after the training through continuous support.
- **Supervision:**
  - Direct supervision during in-person training sessions, particularly during patient simulations.
  - Optional virtual check-ins and email support during the clinical practice period.
- **Adaptation:** Content and exercises are adapted to reflect participants’ clinical contexts and the diverse needs of patients with low back pain, especially during workshop 2 and during clinical practice through support.
- **Scope of Practice Training:** Participants are educated on managing psychosocial factors within their scope of practice and the criteria for referral to other professionals.

**3. Achieving Competency**

- **Competency Assessment:**
  - Feedback on competency is given through supervised simulated patient interactions.
- **Competency Checklist for self-assessment:**
  - EXPLORE
    - Explore beliefs
    - Explore motor behaviour
    - Validation of patient’s experience and strong therapeutic relationship
  - EXPOSE
    - Movement strategies modifications
    - Gradual exposition to feared movements and activities through exercises
    - Exercise variation
  - EXPLAIN
    - Patient centred explanation
    - Check the understanding (teach back method)
    - Propose a clear structure for the sessions, with shared decision on objectives
  - EMPOWER
    - Increase load and progressive exercises
    - Physical activity self-management
    - Management of flare-up

**4. Intervention: Support and Fidelity Assessment**

- **Mentoring:**
  - Post-training mentoring includes one virtual group session for case presentations and ongoing support.
  - Trainers offer optional personalized guidance for implementing training concepts into clinical practice after the training.
- **Fidelity Testing:**
  - A checklist will be completed by the clinician for each patient participating to the study.

7. References

1.	Holopainen R, Simpson P, Piirainen A, Karppinen J, Schütze R, Smith A, et al. Physiotherapists’ perceptions of learning and implementing a biopsychosocial intervention to treat musculoskeletal pain conditions: a systematic review and metasynthesis of qualitative studies. Pain. juin 2020;161(6):1150‑68.

2.	Simpson P, Holopainen R, Schütze R, O’Sullivan P, Smith A, Linton SJ, et al. Training of Physical Therapists to Deliver Individualized Biopsychosocial Interventions to Treat Musculoskeletal Pain Conditions: A Scoping Review. Phys Ther. 1 oct 2021;101(10):pzab188.

3.	Keefe FJ, Main CJ, George SZ. Advancing psychologically informed practice for patients with persistent musculoskeletal pain: Promise, pitfalls, and solutions. Phys Ther. 2018;98(5):398‑407.

4.	Cashin AG, Lee H, Wand BM, Bagg MK, O’Hagan ET, Rizzo RRN, et al. Mechanisms of education and graded sensorimotor retraining in people with chronic low back pain: a mediation analysis. Pain. 27 juin 2023;

5.	Wood L, Bejarano G, Csiernik B, Miyamoto GC, Mansell G, Hayden JA, et al. Pain catastrophising and kinesiophobia mediate pain and physical function improvements with Pilates exercise in chronic low back pain: a mediation analysis of a randomised controlled trial. J Physiother. juill 2023;69(3):168‑74.

6.	Christe G, Benaim C, Jolles BM, Favre J. Changes in spinal motor behaviour are associated with reduction in disability in chronic low back pain: A longitudinal cohort study with 1‐year follow‐up. Eur J Pain. févr 2024;ejp.2245.

7.	Hill JC, Whitehurst DG, Lewis M, Bryan S, Dunn KM, Foster NE, et al. Comparison of stratified primary care management for low back pain with current best practice (STarT Back): a randomised controlled trial. The Lancet. 2011;378(9802):1560‑71.

8.	Caneiro JP, Smith A, Bunzli S, Linton S, Moseley GL, O’Sullivan P. From Fear to Safety: A Roadmap to Recovery From Musculoskeletal Pain. Phys Ther. 2022;102(2):1‑12.

9.	De Baets L, Meulders A, Van Damme S, Caneiro J p., Matheve T. Understanding Discrepancies in a Person’s Fear of Movement and Avoidance Behavior: A Guide for Musculoskeletal Rehabilitation Clinicians Who Support People With Chronic Musculoskeletal Pain. J Orthop Sports Phys Ther. mai 2023;53(5):307‑16.

10.	Bunzli S, Smith A, Schütze R, Lin I, O’Sullivan P. Making Sense of Low Back Pain and Pain-Related Fear. J Orthop Sports Phys Ther. 2017;47(9):1‑27.
