# Supplementary material for: PRIME - A multilevel intervention for low back pain management in primary care: Protocol for an effectiveness-implementation hybrid type 1 cluster randomized controlled trial
Source: Contemp Clin Trials Commun. 2026 Aug 12;53:101679. doi: 10.1016/j.conctc.2026.101679 (PMC13499054; doi:10.1016/j.conctc.2026.101679)
Supplement: Supplementary file 4 [file mmc4.docx]

**Table 1: General practitioners timeline**

| **GENERAL PRACTITIONERS** | **STUDY PERIOD** | | | | | |
| --- | --- | --- | --- | --- | --- | --- |
|  | **Enrolment** | **Allocation** | **Post-allocation** | | | |
| **TIMEPOINT**** | **Pre-randomization** | **Pre-training** | ***Baseline measures*** | ***Prior to patient recruitment*** | ***At the end of training / 2 months after baseline*** | ***12 months after patient recruitment initiation*** |
| **ENROLMENT:** |  |  |  |  |  |  |
| **Eligibility screen** | X |  |  |  |  |  |
| **Informed consent** | X |  |  |  |  |  |
| **Allocation** |  | X |  |  |  |  |
| ***Intervention group:*** |  |  |  |  |  |  |
| ***Training (2x 1h)*** |  |  |  |  |  |  |
| ***Reminders*** |  |  |  |  |  |  |
| ***Emails with clinical pathway*** |  |  |  |  |  |  |
| ***PRIME intervention to patients*** |  |  |  |  |  |  |
| ***Back-PAQ*** |  |  | X |  | X | X |
| ***Implementation questionnaire*** |  |  |  |  | X | X |
| ***Focus Group*** |  |  |  |  |  | X |
| ***Control group:*** |  |  |  |  |  |  |
| ***Usual care*** |  |  |  |  |  |  |
| ***Back-PAQ*** |  |  | X |  | X | X |

**Table 2: Physiotherapists timeline**

|  | **STUDY PERIOD** | | | | | |
| --- | --- | --- | --- | --- | --- | --- |
| **PHYSIOTHERAPISTS** | **Enrolment** | **Allocation** | **Post-allocation** | | | |
| **TIMEPOINT**** | **Pre-randomization** | **Pre-training** | ***Baseline measures*** | ***Prior to patient recruitment*** | ***At the end of training*** | ***12 months after patient recruitment initiation*** |
| **ENROLMENT:** |  |  |  |  |  |  |
| **Eligibility screen** | X |  |  |  |  |  |
| **Informed consent** | X |  |  |  |  |  |
| ***Intervention group:*** |  |  |  |  |  |  |
| ***2-day workshop*** |  |  |  |  |  |  |
| ***PRIME intervention to patients*** |  |  |  |  |  |  |
| ***Back-PAQ*** |  |  | X |  | X | X |
| ***Clinical Vignettes*** |  |  | X |  | X | X |
| ***Implementation questionnaire*** |  |  |  |  | X | X |
| ***Interviews*** |  |  |  |  | X |  |
| ***Focus Group*** |  |  |  |  |  | X |

**Table 3: Patients timeline**

|  |  | **STUDY PERIOD** | | | | | | | | | |
| --- | --- | --- | --- | --- | --- | --- | --- | --- | --- | --- | --- |
|  | **Enrolment** | | **Allocation*** | **Post-allocation** | | | | | | | |
| **TIMEPOINT**** | **First consultation** | | **First consultation** | ***Baseline 1 (after consultation)*** | ***Baseline 2 (2 days)*** | ***10 days*** | ***6***  ***weeks*** | ***3 months*** | ***6 months*** | ***9 months*** | ***12 months*** |
| **ENROLMENT:** |  | |  |  |  |  |  |  |  |  |  |
| **Eligibility screen** | X | |  |  |  |  |  |  |  |  |  |
| **Informed consent** | X | |  |  |  |  |  |  |  |  |  |
| **Allocation** |  | | X |  |  |  |  |  |  |  |  |
| **INTERVENTIONS:** |  | |  |  |  |  |  |  |  |  |  |
| ***PRIME intervention*** |  | |  |  |  |  |  |  |  |  |  |
| ***Usual care*** |  | |  |  |  |  |  |  |  |  |  |
| **ASSESSMENTS:** |  | |  |  |  |  |  |  |  |  |  |
| ***RMDQ*** |  | |  | X |  | X | X | X | X | X | X |
| ***BCCS*** |  | |  | X | X | X | X | X | X | X | X |
| ***NRS*** |  | |  | X | X | X | X | X | X | X | X |
| ***EQ-5D-5L*** |  | |  | X |  |  |  | X | X |  | X |
| ***SBT*** |  | |  | X |  | X | X |  |  |  |  |
| ***WPAI*** |  | |  | X |  | X | X | X | X | X | X |
| ***Back-PAQ*** |  | |  |  | X |  | X |  | X |  | X |
| ***PSEQ-2*** |  | |  |  | X |  | X |  | X |  | X |
| ***BSQ*** |  | |  |  | X |  | X |  | X |  | X |
| ***Reassurance*** |  | |  |  | X |  |  |  |  |  |  |
| ***GRC*** |  | |  |  |  |  |  | X | X |  | X |
| ***Healthcare utilization*** |  | |  |  | X | X | X | X | X | X | X |
| ***Demographics, including SDOH*** |  | |  |  | X |  |  |  |  |  |  |
| ***PREMs (satisfaction)*** |  | |  |  | X |  | X |  | X |  | X |
| ***Qualitative interviews*** |  | |  |  |  |  |  |  | X |  |  |

Back-PAQ: Back Pain Attitudes Questionnaires; RMDQ: Roland–Morris Disability Questionnaire; BCCS: Back capacity and confidence scale; NRS: Numerical Rating Scale; EQ-5D-5L: health-related quality of life; SBT: Start Back Tool; WPAI: Work Productivity and Activity Impairment Questionnaire; PSEQ-2: Pain Self-Efficacy Questionnaire short form; BSQ: Brief screening questions on Fear of movement, Catastrophization, Anxiety, Depression and Expectation to return to work; GRC: Global Rating of Change
